# Stimulus-dependent deliberation process leading to a specific motor action demonstrated via a multi-channel EEG analysis

**DOI:** 10.3389/fnhum.2015.00355

**Published:** 2015-07-02

**Authors:** Sonja Henz, Dieter F. Kutz, Jana Werner, Walter Hürster, Florian P. Kolb, Julian Nida-Ruemelin

**Affiliations:** ^1^Department of Physiological Genomics, Motor Research, Institute of Physiology, Ludwig-Maximilians-University of MunichMunich, Germany; ^2^Research and ConsultingUlm, Germany; ^3^Department of Philosophy IV, Ludwig-Maximilians-University of MunichMunich, Germany

**Keywords:** deliberation, high density EEG, color-word-Stroop task, choice-reaction task, movement intention, Libet, readiness potential, free will

## Abstract

The aim of the study was to determine whether a deliberative process, leading to a motor action, is detectable in high density EEG recordings. Subjects were required to press one of two buttons. In a simple motor task the subject knew which button to press, whilst in a color-word Stroop task subjects had to press the right button with the right index finger when meaning and color coincided, or the left button with the left index finger when meaning and color were disparate. EEG recordings obtained during the simple motor task showed a sequence of positive (P) and negative (N) cortical potentials (P1-N1-P2) which are assumed to be related to the processing of the movement. The sequence of cortical potentials was similar in EEG recordings of subjects having to deliberate over how to respond, but the above sequence (P1-N1-P2) was preceded by slowly increasing negativity (N0), with N0 being assumed to represent the end of the deliberation process. Our data suggest the existence of neurophysiological correlates of deliberative processes.

## Introduction

### Philosophical aspects

This study was conceived and planned in an interdisciplinary frame. The researchers involved work in the area of physics, electrical engineering, neuroscience, and philosophy.

In Libet's seminal study (Libet et al., 1983a), participants were asked to flex their hand whenever they wanted to within a given period of time and to record the time of the decision to flex the hand. Libet called this decision the “conscious act of will,” the time of which the participants had to determine by looking at a fast rotating dot on a clock face (2.56 s per round). Scalp electrodes recorded the participants' electroencephalograms (EEG). Based on the EEG activity, Libet et al. stated that the readiness potential appeared some 550 ms before the actual behavior began. In contrast the participants themselves recorded their decision to flex the hand on average only 200 ms before the so-called motor act. Since the onset of the readiness potential thus preceded the time at which participants reported their decision, Libet concluded that movements in general are caused by unconscious processes in the brain and not by a free decision or, in a broader sense, by free will. Since its first publication in 1983 (Libet et al., 1983a) many surveys of a similar design have followed and have been interpreted similarly (e.g., Haggard and Eimer, 1999; Haggard, 2011).

Although from the very beginning commentators have doubted this justification for this conclusion (e.g., Herrmann et al., 2008). Libet's study became a *locus classicus*, seemingly showing that the results of neuroscientific research are incompatible with the existence of free will. Interestingly enough, Libet himself felt the urge to include the possibility of a veto, i.e., the possibility of stopping a movement already initiated by a readiness potential, in order to save at least a relict of free will. He assumed that the complete dismissal of free will would destroy an essential part of our human self-image and that this should not be given up as long as there is even a faint hope of maintaining some of it. Indeed, experiments show that ongoing EEG activity does not necessarily lead to a motor act—there seems to be the option of “vetoing” a certain behavior that neurophysiologically was already “prepared” by ongoing EEG activity (Haggard and Eimer, 1999; Trevena and Miller, 2010). This raises doubts regarding the causal role of this EEG activity for the respective motor act.

Recent follow-up studies to Libet's experiment suggest that the readiness potential is just what its name suggests—a mere readiness potential—and not the cause of motor action (Trevena and Miller, 2010). It is thus irrelevant whether the mental event thought to be crucial for conscious control of behavior is reported to have taken place before or after the ongoing readiness potential. In essence Trevena and Miller modified Libet's experiment so that the participants had to move either their right or the left hand depending on whether the sign “L” or “R” is shown. The participants were instructed thus: “*At the start of each trial you will see an L or R, indicating the hand to be used on that trial. However, you should only make a keypress about half the time. Please try not to decide in advance what you will do, but when you hear the tone either tap the key with the required hand as quickly as possible, or make no movement at all*.” (Trevena and Miller, 2010, p. 449). The amplitude of the readiness potential was found not to vary according to whether or not participants actually moved their hand after the tone, indicating that the readiness potential cannot be the cause of this motor action. But there is a critical point in the Trevena's and Miller's results: What is the role of the EEG negativity that is ongoing before the tone? Although data were baseline-corrected (for 200 ms based on an interval from 1.3 to 1.5 s before the tone) the negativity remained. Trevena and Miller interpreted that as the involvement with the task, although this does not necessarily explain the temporal correlation of the negativity and the tone: “*[…] as the participants could not predict the time of the tone, this negativity seems to indicate a general ongoing involvement of the participants with the task—perhaps anticipation of the tone […]*.” (Trevena and Miller, 2010). In fact, the tone was produced after a random delay of 2000 + *x* ms, where *x* varied according to an exponential distribution with a mean of 2000 ms. On average the time period between two tones was thus 4 s. This is short enough to generate an expectation in the participants. The question whether this expectation is conscious or should rather be interpreted as a mere adaptive process seems irrelevant here. Our experimental design focuses on the causal role of deliberation and so excludes complications of this type.

The philosophical concept of human responsibility and freedom presupposes that deliberating reasons is causally relevant for what we do (Nida-Rümelin, 2005). The Libet study and its followers, however, exclude deliberation from the start by instruction and therefore cannot show what was claimed to have been shown, namely that deliberation is causally irrelevant. Our experimental study fills this gap by including an element of deliberation in the experimental design, and interestingly enough, makes the causal role of deliberation evident.

### Physiological aspects of motor activation

Electrical potentials, preceding and occurring during voluntary movements have been studied extensively (Vaughan et al., 1968). The initial component, termed N1, is a slow negative shift associated with a dorsiflexion of the wrist and was recorded from the left Rolandic cortex 4 cm from the midline. Later components have been described as positivity (P1), followed by a negativity (N2) which corresponded to the EMG of the wrist muscles, and a final positivity P2, which was assumed to represent the afferent input from joint and muscle receptors. For self-paced movements brain activity can be detected approximately 1000 ms prior to the corresponding electromyographic activity. This brain activity is accepted as the readiness potential (Kornhuber and Deecke, 1964, 1965). The studies of Libet et al. (1983a) were designed initially to detect a temporal relationship between brain activity and the occurrence of conscious realization of the intended movement. Their results were interpreted as showing that motor acts start unconsciously some 300 ms preceding the time of awareness of the intended movement. A more recent study by Haynes' group found that the outcome of a decision should be encoded in brain activity of prefrontal and parietal cortex up to 10 s before it enters awareness (Soon et al., 2008).

Conscious movements result from preceding mental processes. Deliberating and evaluating a number of stimuli lead to the final decision of when and how to move. Such a deliberation process can be evoked by presenting conflicting stimuli with the subject not knowing the movement to be employed for responding to the next stimulus. A conflicting approach was introduced by Stroop (1935) who showed that the interference of conflicting word stimuli on the time for naming 100 colors (each color being the print of a word which names another color) resulted in an increase of 74.3% compared with the normal time for naming colors printed. Desoto et al. (2001) pointed out that conflicting trials require different cortical processing than do non-conflicting trials.

Short latency motor-related activity is found above motor areas in subjects performing a simple motor response to an expected and known visual stimulus. As noted above, in subjects performing a voluntary, self-paced finger movement, cortical electrical activity is found prior to the motor act and has been accepted as the readiness potential (Kornhuber and Deecke, 1964, 1965). Both aspects however, are different from our approach in which subjects do not know either the time of occurrence or the specificity of the upcoming stimulus, that determines the finger to be employed for a corresponding movement. Consequently a mental process is required that deliberates among a number of stimuli leading to an adequate decision for the correct motor act. This is the first out of a series of several studies with the aim to characterize electrophysiological cortical activity preceding a specific motor act, recorded via a multichannel EEG system, and to determine the causal role of a deliberation process represented by these signals. In the present study data recorded primarily from the cortical hand area are shown.

## Materials and methods

### Subjects

A total of 28 students of the Ludwig–Maximilians-University in Munich participated in this study; each of them gave written informed consent prior to the start of the experiment; each was paid € 35.00 for participation in a single 3-h experimental session. Three subjects were excluded for technical reasons. The remaining group (mean age ± SD: 22.9 ± 2.2; 18–27 years) consisted of 13 females and 12 males. All participants had normal or corrected-to-normal vision and had no history of neurological or psychiatric disorders. The local Ethics Committee of the Medical Faculty of the Ludwig-Maximilians-University of Munich was informed of the study.

### Paradigm

Electrophysiological signals preceding a voluntary motor act have been reported in many publications (e.g., Kornhuber and Deecke, 1964; Gilden et al., 1966; Shibasaki et al., 1980; Toma et al., 2002). If the motor act depends on a diversity of stimulus conditions, a deliberation process will precede the decision for a movement. In the current study, subjects had to press buttons with the index finger of the right or left hand, depending on a visual stimulus.

### Stimulation protocol

During the experiment, participants sat comfortably in front of a standard 22″ computer monitor (BenQ GL2250M, 16:9) at a distance of 80 cm. The subjects' hands rested on a table with the index fingers positioned on one button each. The screen showed regular checkerboard fields (24 in *x*-direction and 20 in *y*-direction) with a single field size of 1.43 × 0.96°, respectively. During the experiment, participants had to fix their gaze on a gray fixation point in the center of the screen (0.32°).

#### Visually evoked potential (VEP)

Three types of stimulus-reaction tasks were tested. In the initial series, visually evoked potentials (VEP) were recorded with the reversal of the checkerboard as stimulus. Repetitions (300) were given at an inter-stimulus interval of 750 ms. An additional set of 300 visually evoked potentials (VEP-text) were recorded, during which a text field indicating the name of a color (e.g., “red”) was shown for 300 ms, starting at the time of the reversal of the checkerboard. The size of the text field is provided below in the description of the third series of stimulus reaction task (see deliberation task).

#### Motor task

**Figure 1:**
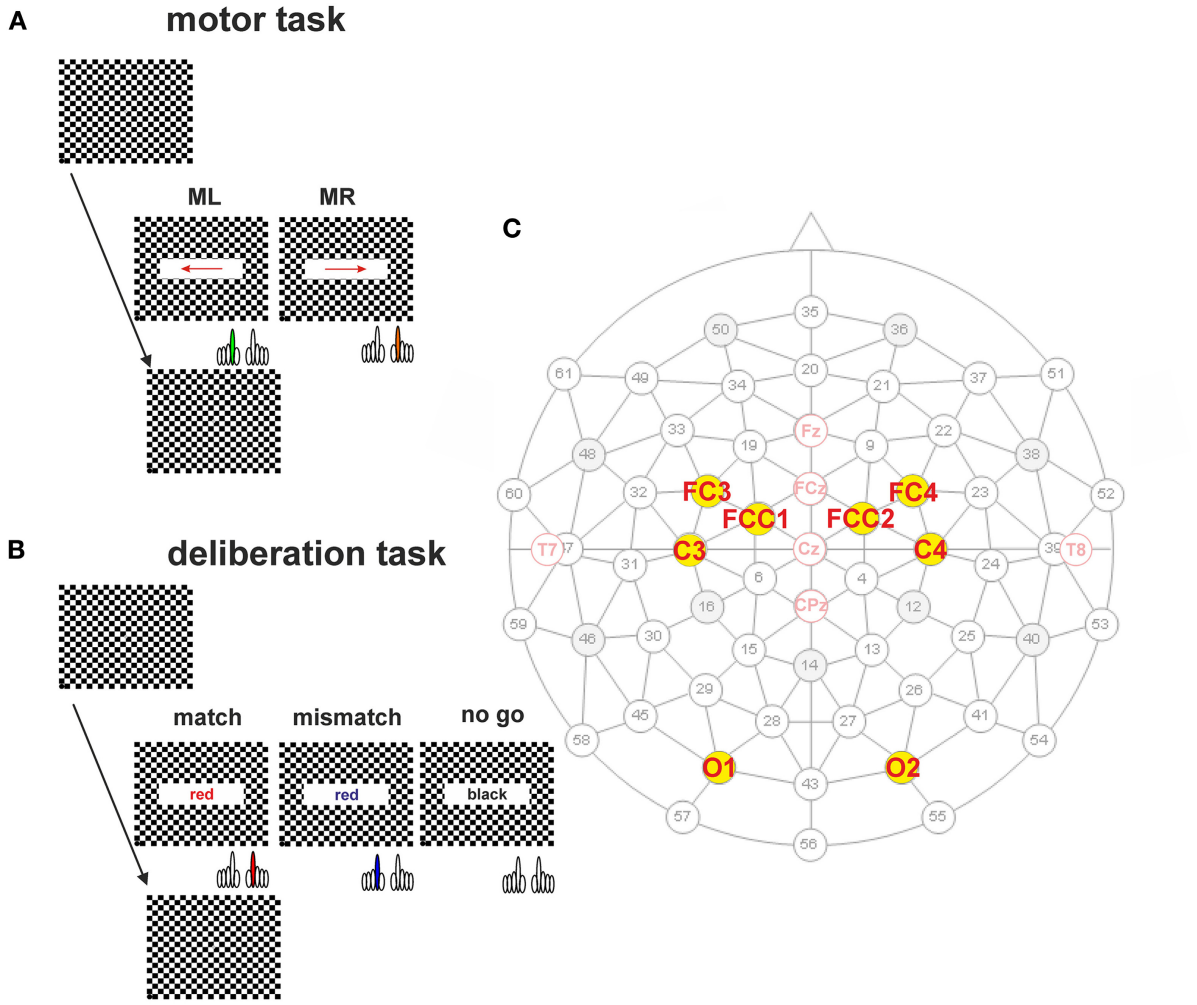
**Protocols of the simple motor task (A) and the deliberation task (B). (A)** Subjects sat in front of a computer monitor showing a checkerboard. At the time of pattern reversal either an arrow (motor task) or a colored text (deliberation task) was shown for 300 ms. The inter-stimulus interval was randomized (12–15 s). The checkerboard pattern reversal was recorded by an opto-electronic sensor situated at the lower left corner of the screen (marked as black dot). **(A)** Subjects were informed about the direction of the arrow prior to the measurement of 50 trials. An arrow pointing to the right had to be responded to with a keypress by the right index finger (orange, *MR*). Another 50 trials are given at the same intervals but the direction of the arrow was reversed and the left index finger (green, *ML*) was used for the keypress. **(B)** During the deliberation tasks a text field showing the written name of a color was shown at the time of pattern reversal. During match-trials the name of the color coincided with the color of the text. Subjects had to respond with a keypress by the *right* index finger (red). During mismatch-trials the name of the color did not coincide with the color of the text. Subjects had to respond with a keypress by the *left* index finger (blue). *No-go-trials* were characterized by the word “*black*” given in a black color on the screen. During these type of trials subjects should not respond by a motor action. **(C)** Schematic representation of the 61-electrode EEG-cap with equidistant electrode positions are marked in black (subdued), and marked in red for electrode positions according to the ten–twenty system (e.g., Jasper, 1958) and the revised version by Jurcak et al. (2007). The electrodes analyzed in this study are marked by yellow dots with solid black circles. The names of the electrodes—written in red—are according to the revised convention (Jurcak et al., 2007).

In the second series, the subjects' reaction time was tested by recording the keypress of the index finger of the right or left hand (Figure 1A, motor task). For this test, each reversal of the checkerboard pattern was combined with the appearance for 300 ms of a right-pointing arrow as stimulus, to which the subject had to respond with the right index finger (Figure 1A, MR), or a left-pointing arrow for the left index finger (Figure 1A, ML). Subjects were aware of the direction of the arrow, during a block of 50 sequential repetitions in each direction at random inter-stimulus intervals of 12–15 s. The size of the field in which the arrow was displayed was 66% of screen width (*x*-direction: 316.8 mm; 21.6°), and 20% of screen height (*y*-direction: 54 mm, 3.86°).

#### Deliberation task

The third series consisted of the actual deliberation task, a type of color-word Stroop-task (Stroop, 1935) which consisted of three blocks of 100 trials each. The deliberation-task conditions occurred at the same time as the reversal of the checkerboard pattern with a text field in the center of the screen (horizontal direction 66% of screen width, and in vertical direction 20% of screen height) as stimulus. Within the text field a color name was printed in color (Figure 1B, deliberation task). When the name of the color coincided with the print color shown, subjects had to press the right button (*match*-trial), if it did not coincide, subjects had to press the left button (*mismatch*-trial). If the color name was “black” and it was shown in black, no buttons had to be pressed (*no-go* trial). The text fields were presented for 300 ms at randomized interstimulus intervals of 12–15 s with a probability of 1/3 for each trial type.

### Data recording

During the whole session, the subject's brain activity was recorded continuously via a computer-assisted 64-channel recording system (Electroencephalograph Neurofax EEG-1200 pro, Nihon Kohden). Integrated in the Neurofax EEG-1200 was a Sony video system (Sony EVI-D70P) for simultaneous recording of the subject's face and facial muscle activity (e.g., frowning). Subjects wore an EEG-cap (Montage No. 10, Easycap GmbH, Herrsching, Germany) with 61 equidistant Ag/AgCl-electrodes (subdued gray numbers and circles; mean ± SD: 37 ± 3 mm, given at a head circumference of 58 cm). The pale red circles in Figure 1C represent the electrode positions according to the clinically used ten–twenty system (e.g., Jasper, 1958; Homan et al., 1987). The electrode positions shown as black-bordered solid yellow circles with clinically used names or revised names (Jurcak et al., 2007) represent the electrode positions analyzed in this study (Figure 1C, in red). The cap was mounted such that the center electrode matched the Cz position. The electrodes were backfilled with an electrolyte gel (Electro-Gel; Electro-Cap International Inc., Eaton, Ohio, USA) such that electrode impedances below 50 kOhm could be achieved. The reference electrode was fixed to the left earlobe, the ground electrode to the right earlobe. Electrophysiological data were sampled at 200 Hz, band pass (0.01–70 Hz) filtered, and stored on the Neurofax EEG-1200 pro computer for off-line analysis. During recording a notch filter was activated only for the data display on the screen.

Stimulation and recording was controlled from a laptop (ASUS Pro, B53E), generating the checkerboard pattern and the temporal random sequence of the stimuli. The checkerboard pattern reversal was recorded by an opto-electronic sensor (photo transistor of OPB 813S) situated at the lower left corner of the screen (Figures 1A,B), thus providing an accurate time reference. For the press-buttons, short-way micro switches were used. The corresponding DC signals, obtained via a custom-built interface, were fed to a microcontroller (Ethernet Atmega 32/644). The microcontroller evaluated the information of the checkerboard reversal, the trial type provided (match, mismatch, no-go), the time between the pattern reversals, the time of the subject's keypress (reaction time), whether the trial type corresponded to the performed key action, and fed them online as DC control signals to the Neurofax EEG-1200 pro.

### Data analysis

Trials with wrong responses (<2%) were excluded from further analysis, as they were artifact-adhesive trials. The EEG waveforms were smoothed using a 0.01–40 Hz bandpass. To reduce the effect of blinks and eye-movements on the EEG activity, an eye-movement correction procedure was employed using an artifact reduction tool (BESA Research analysis software, Version 6.0, BESA, Gräfelfing, Germany). With this software package, temporal sections of 500 ms preceding and 2000 ms following the stimulus could be cut out from the continuous data stream.

Generally, three types of time-locked data are shown: data related to the time at which the stimulus occurred (stimulus-aligned); data related to the time at which the key was pressed (key-aligned) and data related to the maximal negativity (N0) of the EEG activity (N0-aligned) prior to keypress.

**Figure 2:**
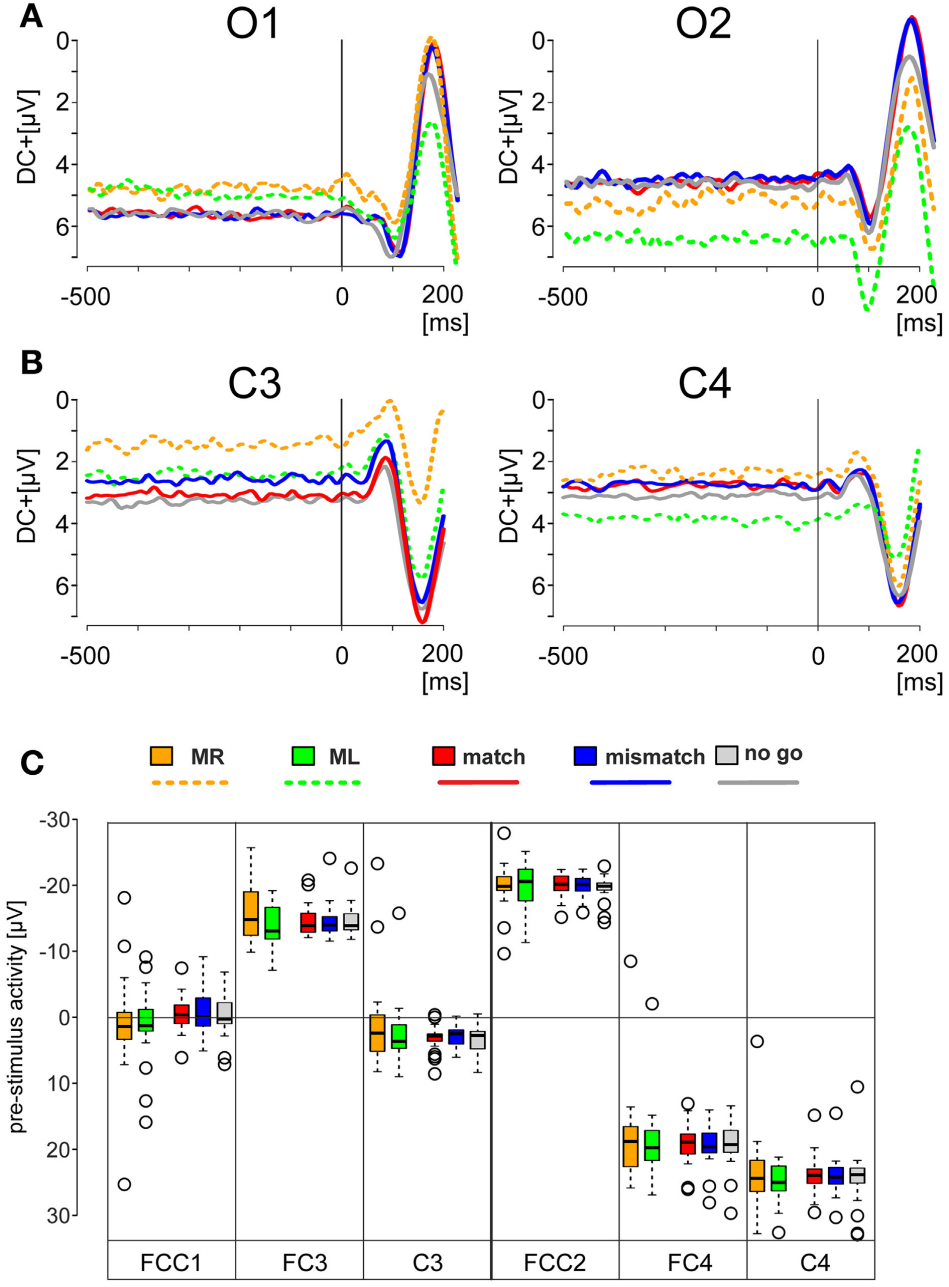
**EEG activity prior to the onset of the visual stimulus. (A,B)** Offset-corrected traces of the averaged potentials recorded from the electrode positions *O1* and *O2* **(A)**, *C3* and *C4* **(B)**. They were obtained during the motor tasks (broken lines) and during match, mismatch, and no-go conditions (solid lines). The traces are color-coded according to the legend below: Motor tasks using the right index finger (orange, *MR*); using left index finger (green, *ML*); match condition (red, *match*); mismatch condition (blue, *mismatch*); no-go condition (light gray, *no go*) conditions. The visually evoked potentials show at these positions a characteristic initial negativity, followed by a large positivity. **(C)** Box plots of the mean values with quartiles of the DC-levels measured for an interval of 400 ms preceding the stimulus onset during the motor and deliberations tasks. Box plots are color-coded as described above. Outliers are represented by open circles.

**Figure 3:**
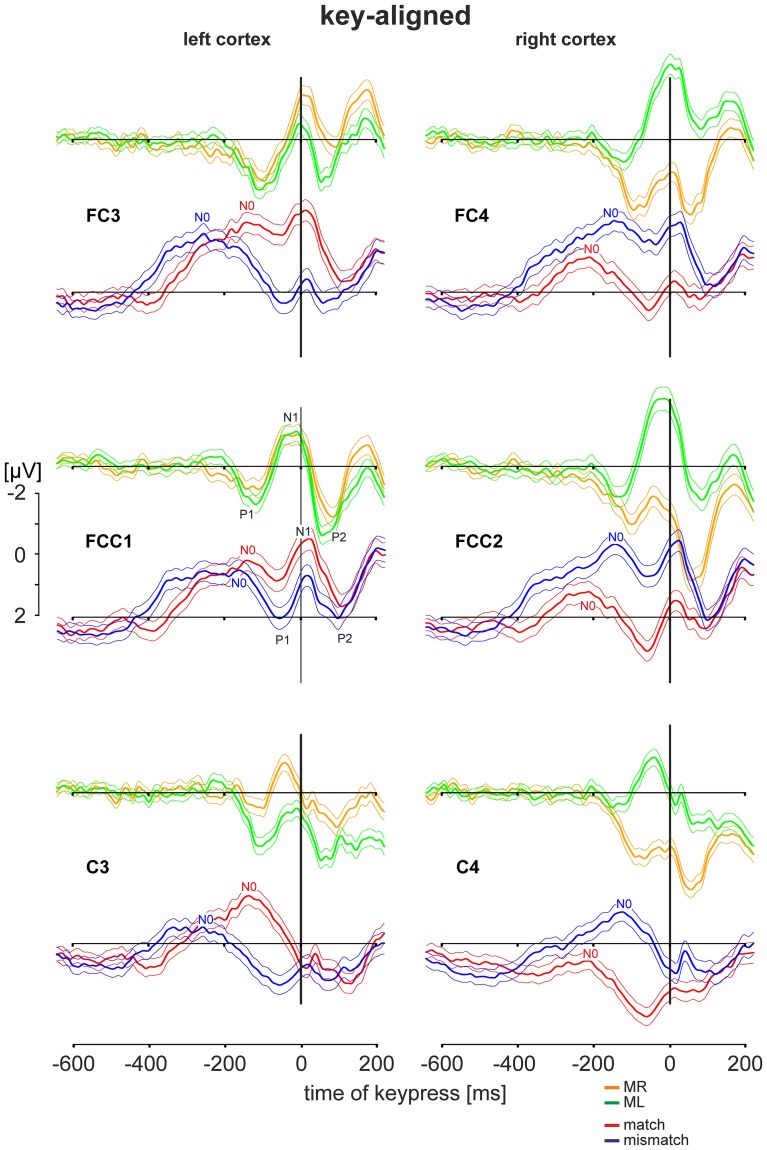
**EEG activities during simple motor tasks and during deliberation tasks**. EEG data were obtained from the *left cortex* (electrode positions *FC3, FCC1, C3)* and from the *right cortex* (electrode position *FC4, FCC2, C4*). (For electrode positions see Figure 1C). For comparison corresponding patterns from the same electrode positions are shown among each other: responses to the motor tasks (performed with the right index finger: *MR*: orange traces; with the left index finger: *ML*: green traces); responses to the deliberation tasks during *match-trials* (red traces) and *mismatch-trials* (blue traces). Patterns are shown as grand averaged EEG responses (thick solid lines) with the 95% confidence limits (thin solid lines) and consist of a sequence of a sharp initial positivity *P1*, followed by negativity N1 and a final positivity *P2* (shown explicitly for the electrode position FCC1). Prior to these sequences additional negativities *N0* are apparent. All patterns are equally scaled from −2 to +2 μV. All EEG activities are aligned to the time of keypress (*key-aligned*) and are shown for the period from −600 ms before keypress to 200 ms after keypress.

Stimulus-aligned data are available during the simple motor task and during match, mismatch and no-go-trials. Key-aligned-data are available during the simple motor task and during match- and mismatch-trials whereas N0-data are available during match and mismatch-trials only. The data were DC-baseline-corrected (except in Figure 2) with the mean value obtained during a 400-ms interval prior to stimulus onset. For this step and for a semi-automatic peak-detection of the EEG data, a custom-written analysis software package with corresponding algorithms has been established in our laboratory based on the language for statistical computing R (Version 3.0.2, 2013-09-25, The R Foundation for Statistical Computing). The data are presented as averaged or grand-averaged responses (e.g., Figure 3).

### Statistical analysis

**Figure 4:**
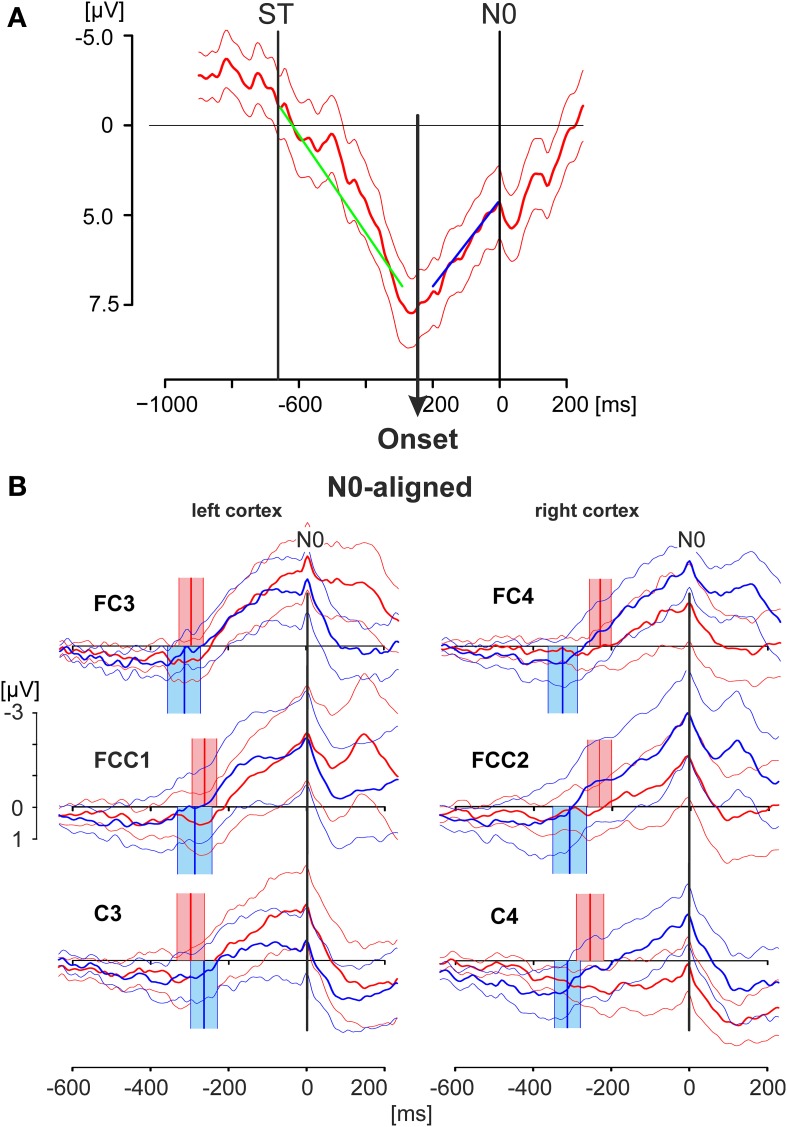
**EEG activity patterns aligned to the negative peak N0. (A)** Averaged EEG pattern (thick solid line; thin solid lines: 95% confidence limits) obtained from electrode position *C3* of a single subject during match-trials showing the method employed to detect the *Onset* of the increasing negativity terminating in *N0*. The onset has been calculated by a regression model describing the data with two different linear splines. The time window starting from the stimulus onset (*ST*) and terminating at the *N0* peak was divided in two intervals at any time point inside the time window. Linear regression analyses were preformed for both intervals (regression lies green and blue). The onset of the increasing negativity was determined in a least-squares setting by that point in time at which the residues of both regressions resulted in a minimal value (see Material and Methods Section). **(B)** The grand averaged EEG activities as displayed in Figure 3 are shown, here aligned to the time of N0. The thick solid lines represent the averaged responses whereas the thin lines indicate the 95% confidence limits of the group mean. Red lines represent responses during match-trials, blue lines those of mismatch-trials. The red and blue blocks cover the time range (95% confidence limits: solid thin lines) of the times of onset (mean values: solid thick lines) as shown in the example in **(A)** for match and mismatch-trials, respectively. Data in **(A)** are shown for the period −1000 to +200 ms and are scaled from −5 to +7.5 μV. Data in **(B)** are shown for the period −600 to +200 ms and are scaled equally from −3 to +1 μV.

**Figure 5:**
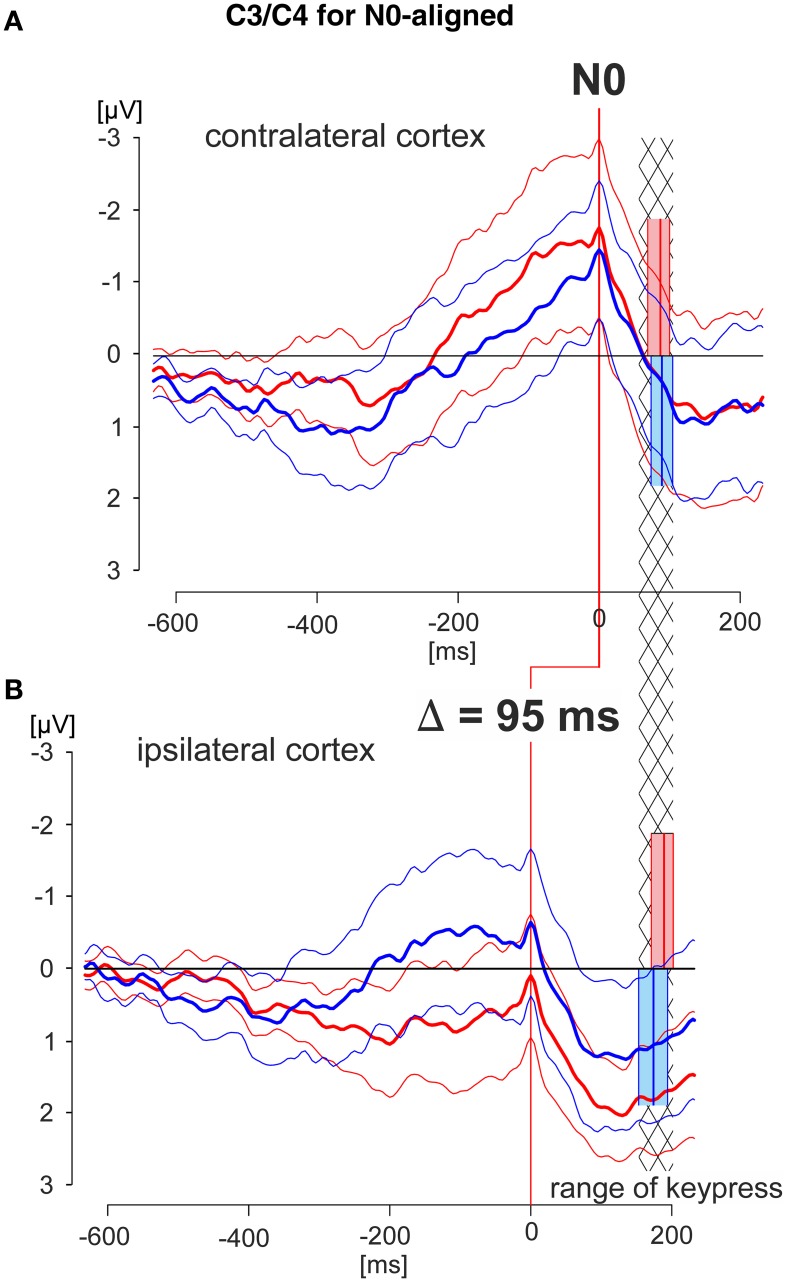
**N0-aligned EEG patterns obtained from C3/C4 recording electrodes of the cortex areas contra- and ipsilateral to the working finger**. Grand averaged EEG patterns were obtained during the deliberation tasks (red lines during match-trials, blue lines during mismatch-trials; solid thick lines: averaged responses, solid thin lines: 95% confidence limits of the group mean). EEG patterns were obtained from the cortex contralateral **(A)** or ipsilateral **(B)** to the working finger, respectively, each of which aligned to *N0*. The red and blue blocks cover the time range (95% confidence limits: solid thin lines) of the times of keypress (mean values: solid thick lines) during match and mismatch-trials, respectively. EEG patterns in **(A)** and **(B)** are aligned in addition such that the times of keypress of the contra- and ipsilateral responses are within corresponding statistical limits (95% confidence limits), indicated by the hatched area. This results in an earlier appearance of the N0 component in the ipsilateral cortex compared with that of the contralateral cortex by 95 ms. Data are shown for a time range 600 ms before to 200 ms after N0, and are equally scaled: (−3 to +3 μV).

Analysis time for stimulus-aligned data is the 500 ms interval preceding the stimulus onset to 200 ms after (Figure 2), for key-aligned data 600 ms preceding keypress to 200 ms after (Figure 3), for N0-aligned data 600 ms prior and 200 ms after (Figures 4, 5). Reaction times were measured from stimulus onset to keypress. All statistical comparisons (hemispherical differences), differences in reaction times, mean values, standard error of the mean (SEM), 95% confidence limits of the mean, were obtained using our laboratory-developed software package based on the language for Statistical Computing R.

For the group analysis, these parameters were treated by appropriate statistical tests, such as unpaired, two-tailed *t*-tests. *P* < 0.05 was assumed to be significant. Due to the high variability of the EEG signals the various components (N0, P1, N1, P2, Figure 3) could not be detected in individual trials but only from the averaged response of each individual electrode position per subject by the semi-automatic peak detection analysis mentioned above.

The onset of the increasing negativity terminating at the N0 component was calculated by a regression model describing the data with two different linear splines. The time window starting from the stimulus onset and terminating at the N0 peak was divided in two intervals at any time point inside the time window. Linear regression analyses were performed for both intervals and the onset of the increasing negativity was determined in a least-squares setting by that point in time at which the residues of both regressions resulted in a minimal value (Meindl et al., 2012). The minimal length of each interval for the regression analyses was set to 50 ms.

For determining whether the maximal negativity N0 is correlated with either stimulus onset or keypress, variances of the durations of the intervals stimulus onset to N0 and N0 to keypress were analyzed by using an adaptation of the Kepner–Randles test (Kutz et al., 2003). This test is a distribution-free test that uses the null hypothesis of bivariate symmetry to detect unequal marginal scales in bivariate populations. The test is insensitive to unequal marginal locations, when this is due to data treatment. Because of its simplicity and power, this statistical test is highly effective. It is based on a Kendall's tau statistic: given two arrays *X*_1_ and *X*_2_, with *X*_1_*_i_* as the interval from stimulus onset to N0 and *X*_2_*_i_* as the interval from N0 to keypress of the *i*-th subject, respectively. The algorithm is as follows (Commenges and Seal, 1985):

Kendall's tau is given by

$$\begin{array}{l} \pi_{n}={\left(\begin{array}{c} n\\ 2 \end{array}\right)}^{-1}\sum_{i<j}\Psi \left[\left(Y_{1j}-Y_{1i}\right)\left(Y_{2j}-Y_{2i}\right)\right]\;with\\ \;Y_{1}=X_{1}-X_{2},\\ \;Y_{2}=X_{1}+X_{2},\\ \Psi \left(t\right)=\{\begin{array}{c} 1\\ 0 \end{array}\begin{array}{c} for\;t>0\\ for\;t\leq 0 \end{array} \end{array}$$

and can be transformed to a normal deviate by

$$\begin{array}{l} \begin{array}{l} Pn=\frac{\sqrt{n}\left(\pi_{n}-0.5\right)}{{\hat{\sigma }}_{n}}\;with\\ {\hat{\sigma }}_{n}^{2}=\frac{4\left(n-2\right)}{n\left(n-1\right)}\sum_{i=1}^{n}{\left[h_{1n}\left(Y_{i}\right)-{\bar{h}}_{1n}\right]}^{2}+\frac{1}{2\left(n-1\right)},\\ {\bar{h}}_{1n}=n^{-1}\sum_{i=1}^{n}h_{1n}\left(Y_{i}\right), \end{array}\\ \begin{array}{l} h_{1n}\left(Y_{i}\right)={\left(n-1\right)}^{-1}\sum_{\begin{array}{c} j=1\\ j\neq i \end{array}}^{n}\Psi \left[\left(Y_{1j}-Y_{1i}\right)\left(Y_{2j}-Y_{2i}\right)\right] \end{array} \end{array}$$

The N0 is correlated with the stimulus onset if the test value of the Kepner–Randles statistic is *Pn* < −1.96, and it is correlated with keypress when *Pn* > +1.96 (α = 5%, two-tailed test).

## Results

The aim of this study was to determine the causal role of a deliberation process that precedes a motor act. Electrophysiological data from a group of 25 young, right-handed, healthy subjects showed a neurophysiological correlate of deliberation that precedes the decision to act. Besides a simple motor task, characterized by pressing one of two buttons, dependent on the stimulus, with either the right or left index finger, a deliberation task i.e., a color-word Stroop task (Stroop, 1935) was employed. For the latter the index finger to be employed depended on the assessment of the information provided on the monitor screen. The present findings represent the initial part of a series of studies and focuses on EEG activities above the hand regions of the primary and the pre-motor cortex only. The EEG activities were analyzed during simple motor tasks and during deliberation tasks (Figure 3).

### Reaction time analysis

Subjects were tested for the basic motor reaction time and for the reaction time during the deliberation task. Since for both tasks a visual stimulus (pattern reversal with an arrow or a colored embedded word, respectively) was employed, visually evoked potentials represent a first non-motor response. Consistent with clinical terminology the latencies (±SEM) of the P100 recorded from O1 during the motor task with arrow pointing right was 114.0 ± 4.6 ms and that with the arrow pointing to the left 110.8 ± 5.2 ms. The corresponding latencies obtained from O2 were 108.8 ± 4.4 and 105.6 ± 3.5 ms (Figure 2A, Table 1). During the deliberation task (match trials) latencies obtained from O1 were 112.6 ± 3.5 ms and during mismatch trials 114.0 ± 3.2 ms. Latencies from O2 were 103.0 ± 3.9 ms during match trials and 101.6 ± 3.2 ms during mismatch trials (Figure 2A, Table 1). These latencies were not significantly different during motor task trials when right- and left-pointing arrows or during match and mismatch trials (Table 1).

**Table 1:** **Reaction times between stimulus onset to VEP and keypress**.

**Reaction times from visual stimulus to VEP and keypress**									
**Condition**	**VEP (O1)**			**VEP (O2)**			**Keypress**		
	**Mean ± SEM [ms]**	**Median**	***p***	**Mean ± SEM [ms]**	**Median**	***p***	**Mean ± SEM [ms]**	**Median**	***p***
**MOTOR TASK**									
Arrow right	114.0 ± 4.6	110.0		108.8 ± 4.4	110.0		264.6 ± 7.9	260.3	
Arrow left	110.8 ± 5.2	105.0		105.6 ± 3.5	105.0		272.3 ± 9.3	271.4	
Delta	3.2	5.0	0.65	3.2	5.0	0.57	−7.7	−11.2	0.53
**DELIBERATION TASK**									
Match	112.6 ± 3.5	115.0		103.0 ± 3.0	105.0		706.5 ± 20.5	704.0	
Mismatch	114.0 ± 3.2	115.0		101.6 ± 3.2	100.0		770.2 ± 19.0	779.2	
Delta	−1.4	0.0	0.77	1.4	5.0	0.75	−63.7	−75.2	<0.05

*Reaction times are given in ms for the motor task and for the deliberation. During both, the latencies of the visually evoked potential **(VEP)**, obtained from electrodes **O1** and **O2** (Figure 1C) are provided. During motor task conditions the arrow pointed either to the right **(arrow right)** or to the left **(arrow left)**. During the **deliberation task** reaction times are provided for **match** and **mismatch**-trials. **Mean** latencies, **SEM**, and **median** are given. The probability **P** is calculated for the differences **(delta)** in latencies*.

The basic motor reaction time was measured in blocks of 50 trials (right index finger first, see Material and Methods). The mean latency for the right index finger was 264.6 ± 7.9 ms and was not significantly different from that for the left index finger (272.3 ± 9.3 ms). The reaction times during the deliberation task were clearly longer. For match conditions (right index finger) the mean reaction time was 706.5 ± 20.5 ms and for mismatch conditions (left index finger) 770.2 ± 19.0 ms (Table 1). These values are significantly (*P* < 0.05) different (Table 1). This cannot be explained by handedness. To familiarize themselves with the deliberation task, subjects performed 50 learning trials resulting in reaction times that were clearly longer (approximately by 60 ms) than those obtained during the following deliberation trials (Table 1).

### Pre-stimulus EEG activity

A change in EEG activity prior to the stimulus onset may be interpreted as evidence for an ongoing conscious or even subconscious process. To demonstrate that the EEG activity recorded is not affected by such processes the pre-stimulus EEG activity was also studied. Grand averages obtained for simple motor tasks (traces with broken lines in Figures 2A,B) and for the deliberation tasks (traces with solid lines in Figures 2A,B) were constructed for an analysis time ranging from 500 ms prior to, and 200 ms after stimulus onset (*t* = 0 ms, Figures 2A,B). The traces are color-coded according to the legend below the grand averages (Figures 2A,B). Each of the five EEG responses was recorded from motor areas obtained from electrode C3 and C4 of 25 subjects. These averaged responses (Figure 2B) were offset-corrected but scaled identically. The distributions of the mean values of the pre-stimulus DC-offsets—color-coded as well—are given as box plots in Figure 2C. The DC-offsets preceding the stimulus onset however, cannot be related to the subjects' upcoming decision.

### Key-aligned cortex activity during a simple motor task and during the deliberation task

The EEG activities during the simple motor task and the deliberation task are shown for the pre-motor areas (electrode positions FC3, FCC1, and FC4, FCC2) and for hand motor areas (electrode positions C3 and C4). The grand averaged activities with the corresponding 95 % confidence limits of the mean are shown for an analysis time ranging from 600 ms prior to and 200 ms after keypress with *t* = 0 as the time the key was pressed (key-aligned). In each set two averaged responses are superimposed: Responses obtained during the simple motor task performed with the right finger (MR, right button pressed) are in orange and those with the left finger (ML, left button pressed) are shown in green. For comparison, data sets from the same electrodes obtained during the deliberation task are in red (match conditions, right button pressed) and in blue (mismatch conditions, left button pressed). All averaged responses are equally scaled (−2 to +2 μV, Figure 3).

### Activity during the simple motor task

During the simple motor task performed with the *right index finger* (orange traces in Figure 3) a sequence of three sharp peaks were observed above the left (i.e., the contralateral) cortex. The first peak, a positivity (P1, e.g., Figure 3, FCC1), was maximal approximately 100 ms prior to keypress. P1 was followed by a negativity (N1) close to the time of keypress, earliest at position C3 and latest and maximal at FC3 (Table 2). The final positivity (P2) occurred approximately 90 ms after keypress and was maximal at FCC1 (exact times for each electrode and each peak amplitude are provided in Table 2).

**Table 2:** **Results from the peak analysis of characteristic potentials obtained during simple motor tasks and deliberation tasks**.

		**Electrode**										**P1**		**N1**		**P2**	
												**[ms]**	**[μV]**	**[ms]**	**[μV]**	**[ms]**	**[μV]**
Right index fin ger (orange)	Cx le ft	C3										−107.8 ± 1.6	1.6 ± 0.1	−22.8 ± 1.8	−2.3 ± 0.1	85.0 ± 2.0	2.5 ± 0.1
		FC3										−89.2 ± 1.8	2.7 ± 0.1	13.4 ± 1.3	−2.6 ± 0.1	93.0 ± 1.6	1.3 ± 0.1
		FC1/C1										−106.4 ± 2.0	2.1 ± 0.1	−9.0 ± 1.6	−3.0 ± 0.1	89.4 ± 1.8	2.9 ± 0.2
	Cx right	C4										−82.0 ± 1.3	3.0 ± 0.1	−7.0 ± 1.5	0.1 ± 0.1	71.8 ± 1.3	3.8 ± 0.1
		FC4										−82.8 ± 1.5	3.2 ± 0.1	0.0 ± 1.6	−0.8 ± 0.1	69.2 ± 1.2	3.1 ± 0.2
		FC2/C2										−91.2 ± 1.8	3.2 ± 0.1	−12.6 ± 1.7	−1.0 ± 0.1	83.6 ± 1.7	4.9 ± 0.2
Left index finger (green)	Cx left	C3										−69.6 ± 0.7	4.1 ± 0.1	−6.0 ± 0.7	−1.0 ± 0.1	74.0 ± 0.8	3.0 ± 0.1
		FC3										−71.4 ± 0.7	3.7 ± 0.1	0.2 ± 1.1	−1.8 ± 0.1	75.8 ± 1.0	2.5 ± 0.1
		FC1/C1										−72.6 ± 0.7	3.5 ± 0.1	−9.4 ± 0.8	−2.6 ± 0.1	76.4 ± 0.7	3.4 ± 0.1
	Cx right	C4										−74.0 ± 0.6	2.0 ± 0.1	−16.0 ± 0.6	−3.3 ± 0.1	75.2 ± 1.0	2.0 ± 0.1
		FC4										−72.8 ± 0.7	2.4 ± 0.1	−4.2 ± 0.8	−4.3 ± 0.1	81.2 ± 0.9	0.6 ± 0.1
		FC2/C2										−75.6 ± 0.7	2.8 ± 0.1	−10.8 ± 0.7	−4.1 ± 0.1	80.6 ± 1.0	2.3 ± 0.1
			**Delib Onset**			**N0**						**P1**		**N1**		**P2**	
		**Electrode**	**[ms]**			**[ms]**			**[μV]**			**[ms]**	**[μV]**	**[ms]**	**[μV]**	**[ms]**	**[μV]**
Match (red)	Cx left	C3	−292.0 ± 17.9			−85.4 ± 7.3			−1.8 ± 0.6			2.8 ± 1.4	0.8 ± 0.1	45.6 ± 1.6	−0.4 ± 0.1	125.4 ± 1.6	1.7 ± 0.1
		FC3	−297.4 ± 15.8			−111.2 ± 7.9			−2.9 ± 0.5			−49.8 ± 0.9	−1.4 ± 0.1	20.8 ± 0.9	−3.2 ± 0.1	118.6 ± 1.2	0.2 ± 0.1
		FC1/C1	−261.4 ± 16.2			−142.4 ± 9.6			−2.3 ± 0.8			−54.2 ± 1.5	−0.7 ± 4.5	28.8 ± 0.9	−3.1 ± 0.2	113.6 ± 1.2	0.1 ± 0.2
	Cx right	C4	−248.9 ± 17.8			−186.6 ± 7.9			0.1 ± 0.4			−65.8 ± 1.3	2.7 ± 0.1	32.0 ± 1.5	0.9 ± 0.1	96.0 ± 1.4	2.0 ± 0.1
		FC4	−227.7 ± 14.0			−167.4 ± 8.7			−1.4 ± 0.6			−49.6 ± 1.2	0.9 ± 0.1	38.4 ± 2.2	−1.1 ± 0.1	103.8 ± 1.7	0.5 ± 0.2
		FC2/C2	−224.6 ± 15.5			−200.4 ± 11.5			−1.6 ± 0.7			−52.2 ± 1.0	1.4 ± 0.1	31.2 ± 1.1	−1.0 ± 0.1	96.8 ± 1.1	0.9 ± 0.1
Mis-match (blue)	Cx left	C3	−260.6 ± 17.4			−173.6 ± 10.4			−0.6 ± 0.5			−48.0 ± 1.5	1.8 ± 0.1	25.0 ± 1.4	0.2 ± 0.1	97.4 ± 1.7	1.7 ± 0.1
		FC3	−314.6 ± 21.4			−164.4 ± 11.0			−2.1 ± 0.5			−41.0 ± 1.4	0.9 ± 0.1	26.6 ± 1.3	−0.7 ± 0.1	103.8 ± 1.5	1.0 ± 0.1
		FC1/C1	−285.6 ± 22.5			−157.4 ± 10.4			−2.2 ± 0.8			−41.6 ± 1.1	0.6 ± 0.2	37.4 ± 1.1	−1.7 ± 0.1	114.4 ± 1.4	0.6 ± 0.1
	Cx right	C4	−307.4 ± 16.8			−88.8 ± 8.1			−1.5 ± 0.5			18.2 ± 1.0	1.2 ± 0.1	62.8 ± 1.0	−0.3 ± 0.1	122.2 ± 1.3	1.5 ± 0.1
		FC4	−323.5 ± 19.1			−138.6 ± 10.0			−2.7 ± 0.4			−47.0 ± 1.7	−1.0 ± 0.1	31.2 ± 1.6	−3.0 ± 0.1	127.2 ± 1.4	0.4 ± 0.1
		FC2/C2	−301.0 ± 21.8			−125.8 ± 9.3			−3.0 ± 0.7			−38.6 ± 1.4	−0.7 ± 0.1	32.4 ± 1.0	−2.9 ± 0.2	121.6 ± 1.3	0.6 ± 0.1

*The upper part of the table shows peak mean latency values and SEM for the motor task, the lower part those of the deliberation task. Values are given for the first positivity P1, the negativity N1 and the second positivity P2. For the deliberation task an additional preceding negative peak N0 and the Onset of the negativity terminating in N0 are provided. Temporal values are given in ms. Peak times are related to the time of keypress. Negative values represent times preceding the time of keypress whereas positive values those occurring after keypress. The interval Onset gives the temporal difference between onset and N0. Amplitudes are given in μV and represent the difference to base line corrected values. The color name given in brackets is related to the EEG recordings in Figure 3. Electrode position are according to the ten-twenty system (e.g., Jasper, 1958) on the left or right cortex (Cx) and between FC1 and C1, expressed as FCC1 and FC2 and C2, expressed as FCC2 (Jurcak et al., 2007); (see Figure 1C)*.

During the same movements (right index finger) a corresponding sequence (P1-N1-P2) was observed also above the right (ipsilateral) motor areas (Figure 3, orange, right column), however, all components showed amplitudes, shifted to clearly more positive values (maximal at FCC2, Table 2, Figure 3). It must be emphasized that the numerical mean values given in Table 2 need not necessarily match completely with the peak values in the averaged responses (Figure 3).

Traces related to the movements of the *left index finger* (Figure 3, green, right column) showed in the corresponding contralateral (right) cortex areas (electrodes FC4, FCC2, C4) similar sequences consisting of three sharp peaks (P1, N1, P2): P1 74 ms preceding keypress, N1 close to the time of keypress, and P2 approximately 80 ms after keypress (Table 2). Correspondingly, N1 occurred at C4 prior to and at FC4 immediately before time of keypress (Table 2). The N1 peaks were larger at FCC2 and at FC4 compared with those N1 peaks observed above the corresponding left cortex (Table 2).

As was the case for the movements of the right index finger for the right (ipsilateral) motor areas, the movements of the *left index finger* evoked a similar sequence of three sharp peaks within the ipsilateral (left) cortex (electrodes FC3, FCC1, C3, green, Figure 3, left column). The duration of this sequence however, was shorter (P1 later and P2 earlier) than that of the sequence during movements of the right index finger (MR-trials) in that part of the cortex.

### EEG activity during the deliberation task

During the deliberation task subjects were unaware of the upcoming stimulus and thus, also unaware of whether a movement action was going to be required and, if so, which finger was to be moved. The EEG activity patterns obtained during this type of task were completely different to those recorded during simple motor tasks (Figure 3, red and blue traces). The sequence of the peaks during the deliberation tasks was clearly less sharp than during the simple movement and consisted of at least four peaks. There was an early and long lasting increasing negativity with a final peak, termed N0, preceding the P1-N1-P2 sequence.

During *match-trials* (Figure 3, red), N0 peaks occurred in the *contralateral* (left) cortex at all electrodes (FC3, FCC1, C3) approximately −112 ms before keypress (earliest at position FCC1, latest at C3; Table 2). During *match-trials* the earliest N0 responses in the *ipsilateral* (right) cortex at electrodes (FC4, FCC2, C4) was observed approximately −185 ms before keypress.

The corresponding N0 peaks during *mismatch-trials* (Figure 3, blue) occurred in the *contralateral* (right) cortex at all electrodes (FC4, FCC2, C4) approximately at the same time (earliest at position FC4, latest at C4; Table 2). In the *ipsilateral* (left) cortex the N0 peaks occurred within a similar time range (approximately −165 ms), but earlier than the N0 potential during match-trials (−112 ms, see above).

Applying the Kepner–Randles test (Kutz et al., 2003) to determine whether N0 was related to the stimulus onset or to keypress showed a significant relationship between N0 and keypress.

The interpeak intervals P1-N1 and N1-P2 were consistent in both the simple and the deliberation task. The P1-N1 intervals were 75.9 ± 4.0 ms for the motor task and 73.3 ± 4.7 ms for the deliberation task, the N1-P2 interval were 86.6 ± 3.1 ms for the motor task and 77.4 ± 3.8 ms for the deliberation task, indicating that these components can be assumed to be independent of the deliberation process (Table 2). Since the N0 components were observed during the deliberation task alone they were analyzed further.

In the first step the match and mismatch-trials were aligned to the N0 component resulting in patterns shown in Figure 4B. N0 appeared as a very sharp and narrow peak at the end of a long-lasting, continuously increasing negativity, independent of the electrode position and independent of match-(red) or mismatch-(blue) trials (Figure 4B). Because of the variability of the N0 peaks with respect to the time of keypress the components P1, N1, P2 are less clear in Figure 4B than those shown in Figure 3, although they are still recognizable (i.e., Figure 4B, FCC1).

In the second step the onset of the increasing negativity, terminating in N0, was calculated. The procedure is illustrated in Figure 4A and is described in detail in the Materials and Methods Section. The data represent the N0-aligned averaged match-trials including the 95 % confidence intervals of the mean and were taken from a single subject at the electrode position C3. The regression analysis, based on different splines of two intervals—one of which starting at the stimulus onset (−663.3 ms) and the other terminating in N0—resulted here in an onset of −245.0 ms before N0.

The scattering of the onsets at the group level (Table 2, Figure 4B) is expressed by their corresponding mean values and the 95% confidence limits of the mean, given as colored blocks, with red blocks related to match-trials and blue blocks related to mismatch-trials. During match-trials the durations were shorter in the ipsilateral (right) cortex than those in the left cortex (Figure 4B). This aspect is similar for the durations during mismatch-trials which were somewhat shorter on the ipsilateral (left) cortex. The shortest duration was found during match-trials at position C4 and the longest duration during mismatch-trials at position FC4 (Table 2).

In the third step N0-aligned data, obtained from the contra- and ipsilateral cortexes (positions C3 and C4) were compared at the group level (Figure 5). As a reference (timing) point for the comparison the time of keypress was selected. The time ranges of keypress are illustrated in red and blue blocks whereas the temporal overlap of all blocks is represented by the hatched area (Figure 5). Independent of the trial type, the N0 component occurred 95 ms earlier in the ipsilateral cortex than in the contralateral cortex, indicating different temporal processing in the hemispheres, depending on whether it is related to a moving or to a not-moving extremity.

## Discussion

### Comparison of tasks

The deliberative element of decision in this study was realized as a required evaluation of short-lasting visual stimuli leading to a decision for releasing adequate motor commands. The latencies of the field-size-dependent checkerboard-evoked VEP were within the clinically typical range including side difference (95–115 ms Bach et al., 2005). The neurophysiological correlates of the deliberative element preceded the decision to act and both precede the final motor act. To distinguish between the pure motor activity and a mental deliberative process subjects had to perform identical motor acts during a simple motor task and during the deliberation task (Stroop, 1935). For the simple motor task subjects were aware of the type of stimulus, the direction of the arrow, and the finger to be employed after the upcoming stimulus. The only parameter they did not know was the time of occurrence of the stimulus. During the deliberation task subjects were unaware of all of these parameters and had to evaluate the complete stimulus configuration.

Stroop (1935) reported a phenomenon occurring during conflicting mental processes. Actions learnt extensively and used for a long time run automatically and rapidly whereas comparable, but unfamiliar, actions require higher attention and thus require more time for their performance. In this context the deliberation task, with conflicting color-word stimuli employed in the current study, showed that matching trials had significantly shorter reaction times (699.8 ms) than mismatching trials (760.3 ms, Table 1). This effect cannot be related to the subjects' handedness, since in the simple motor task, in which subjects were aware of the upcoming stimulus, reaction times were clearly shorter and not significantly different between both hands (right index finger: 258.9 ms; left index finger: 264.3 ms; Table 1). This is in agreement with Trevena and Miller (2010) who also found no difference in tone-induced mean reaction times between both hands. Matsuhashi and Hallett (2008) reported EMG-based reaction times (mean ± SD: 170.0 ± 40 ms) in subjects who were instructed to perform right index finger extension as quickly as possible every time they heard the tone. In our approach there was no such necessity for being that fast and thus our values are within a comparable range.

### Pre-motion cortical activity

Although a specific stimulus-related EEG activity preceding the stimulus was not observed in any of our three trial types—apart from different constant levels of DC-components (Figure 2)—unspecific expectancy cannot be excluded. This is insofar in agreement with Trevena and Miller (2010), who found a small stimulus-preceding negativity, regardless of whether subjects decided to move or not to move. These authors interpreted their negativity as an ongoing involvement with the task, possibly associated with a sustained attention, but not as a specific electrophysiological sign for the preparation of the upcoming movement. The decision to “move” is comparable to our match- or mismatch-trials, the decision “not to move” to our no-go-trials.

The absence of specific EEG activity preceding the stimulus, as in our study, speaks in favor of an assumed, but not yet started, sequence consisting of a specific stimulus-related mental deliberation process, followed by the decision to move and finally the motor act itself. Activity prior to stimulus presentation, and thus before subjects are able to decide which button to press, does not specifically determine behavior but reflects a general expectation (Herrmann et al., 2008). Activity prior to a volitional movement was observed in Libet-related studies (e.g., Libet et al., 1983b; Keller and Heckhausen, 1990; Libet, 1993, 2002; Haggard and Eimer, 1999) which however, were designed to find a temporal relationship between the conscious perception of an intended movement and the corresponding electrophysiological signals that clearly preceded the awareness. This differs from our experimental approach.

### Motor task related cortex activity

The sequence of P1-N1-P2 potentials was found during both the simple motor task and during the deliberation task, implying that this sequence is related to execution of the final motor program. The P1 component was observed prior to the time of keypress, the N1 approximately at that time, and the P2 afterwards (Figure 3). The interval P1-N1 was approximately 75 ms. P1 may thus be related to the pre-motor positivity (PMP) in subjects performing volitional finger movements, as described in detail by Deecke et al. (1976). Correspondingly, the N1 (Figure 3) can be related to the peak maximum potential observed during these types of movements. The late P2 in our study is assumed to be proprioceptively evoked and may represent the feedback signal (Figure 3). The three-component sequence P1-N1-P2 in our study coincides with the three later components P1-N2-P2 of a complex of four potentials (“N1-P1-N2-P2”) reported by Vaughan et al. (1968).

### Slowly increasing negativity in the cortex

During the deliberation task slowly increasing surface negativity, termed here N0, preceded the above mentioned sequence of three components. Slowly increasing negative potentials have been reported in the literature under various experimental conditions. During voluntary, active hand or foot movements a slowly increasing surface negative cortical potential, termed the readiness potential (RP), has been described by Kornhuber and Deecke (1964, 1965). The initial part of the RP was analyzed subsequently by Shibasaki et al. (1980) and by Barrett et al. (1986) and characterized as consisting of two (RP-NS' i.e., terminal negative slope) or three components (RP-IS'-NS' with IS' intermediate shift), respectively, with IS' and NS' showing steeper slopes. These negativities have different topographic properties. The genuine RP is both wide and symmetrically distributed above the fronto-central parts of the brain (Kornhuber and Deecke, 1965). The IS' was also bilateral, whilst the NS' had a maximum slope more focally confined above central regions in contralateral electrodes (Barrett et al., 1986). These results have been confirmed in a fMRI study combined with a dipole source analysis (Toma et al., 2002).

An electric sign of sensorimotor association and expectancy was first reported by Walter et al. (1964) who analyzed the responses to an auditory stimulus (click, conditional stimulus) and to a sequence of visual flicker (imperative stimulus). The resulting evoked potentials showed typical shapes, with only marginal changes when the flicker was provided 1 s after the click. A characteristic increasing negativity (contingent negative variation, CNV) was observed between both stimuli when subjects were asked to terminate the second stimulus (flicker) by pressing a button. The CNV was independent of the sequence, either click first followed by flicker or vice versa. The crucial aspect was the expectancy for the second stimulus, which had to be responded to by a motor action. Like the RP, the CNV has been separated into an early and a late component (Connor and Lang, 1969). The early CNV may reside in the frontal lobes whereas the late CNV is observed over primary motor and over the supplementary/cingulate areas, as shown by high-resolution spatiotemporal statistics and current source density analysis (Cui and Deecke, 2000). It should be emphasized that the generators assumed for the late CNV differ from those responsible for the RP (Ikeda et al., 1994; Cui and Deecke, 2000).

A slowly increasing negative potential has been also described by Vaughan et al. (1968). Termed N1, it preceded the later components P1-N2-P2 as mentioned above. The corresponding movements were self-paced and not responses to discrete external signals. The maximal amplitude of the N1 component was found approximately 4 cm from the midline of the contralateral central region. It was assumed to be specifically related to the physiological processes associated with preparation for movement rather than to expectancy, implying a broader preparatory phenomenon, not necessarily dependent upon anticipation of a motor act (Walter et al., 1964). This potential component, however, was observed in self-paced movements performed in 3–4 s intervals and thus, were obtained under different experimental approaches. The generators responsible for may share also those producing the N0 component in our study.

### Functional aspects of the negativity N0

The individual negativities are assumed to be produced by different sources in the brain and thus need not necessarily be identical to the N0 potential of the current study. In the study of Kornhuber and Deecke (1965) subjects decided completely volitionally the point in time for performing a hand/finger movement. A sequence of cerebral potentials during bilateral simultaneous voluntary self-initiated index finger extensions, similar to that shown in the present study (Figures 3, 4B) can be seen in Deecke et al. (1999), Figure 2B. In contrast, in our study subjects were asked to perform an index finger movement following a visual stimulus. The decision of which index finger has to be employed implies a stimulus-dependent deliberation.

The late CNV was originally assumed to be evoked by a second, expected, ***external*** stimulus. Recent studies have indicated that it also represents response preparation, since the amplitude of the CNV is modulated by specific movement parameters related to the movement direction (Mackay and Bonnet, 1990), to the duration of the forthcoming movement (Vidal et al., 1995) and to covert processes underlying preparatory effects as well as their locus within the processing chain (Leuthold et al., 2004). Based on this interpretation of the CNV the N0 component in our study is assumed to represent a corresponding *internal* stimulus, triggering a motor action. Thus, the N0 represents the end of a required deliberation process with the decision of activating a corresponding index finger.

### Putative onset and duration of the deliberation process

The calculated onset of the increasing negativity can be interpreted as an estimate of the initiation of the deliberation process (Figure 4). Consequently, based on the assumption that N0 represents the end of the deliberation process, the interval between the calculated onset of the increasing negativity up to N0 may represent the duration of the deliberation process (see values in Table 2). In experiment 1 of Trevena and Miller (2010) participants had to perform tasks according to the conditions: “Always move” and “sometimes-move.” The reaction times were 322 ms for the condition always-move, and 355 ms for the condition sometimes-move. The authors suggested that it takes about 33 ms to decide whether or not to move. That study differs somewhat from our experimental approach. During “sometimes-move” trials the hand to be employed for the movement was shown explicitly by a letter (R, L) for 1 s on the screen whereas in our case subjects had to evaluate the deliberation task information which was visible for 300 ms only. These differences may be due for longer time to “decide” which included in our study a time preceding the decision by deliberation.

### Functional implications of N0 timing

It is important to note that, in general, N0 components were observed during both match-trials and mismatch-trials and above both contralateral and ipsilateral hemispheres. The N0 potentials occurred later, i.e., closer to the time of keypress for the *working* index finger, i.e., contralateral hemisphere for match-trials and ipsilateral hemisphere for mismatch-trials (Figure 3). The latest N0 components (i.e., 80–90 ms before keypress) were found during match-trials in position C3 and for mismatch-trials in position C4, respectively (Figure 3). Conversely, N0 components of the *non-working* index finger occurred early before keypress (Table 2). This aspect is convincingly shown in Figure 5 with N0-aligned data obtained from these positions representing either as contralateral (upper half of Figure 5) or ipsilateral responses (lower half of Figure 5) to the finger movement. Shifting N0-aligend data sets, such that the ranges of times of keypress overlap completely (Figure 5, hatched column), results in a difference of 95 ms between ipsi- and contralateral (Figure 5). These observations can be explained by the assumption that there is an initial bilateral activation of both hand areas as long as the deliberation is not completely terminated (Desoto et al., 2001; Szucs et al., 2009). After the deliberation process the ipsilateral finger-related cortex areas have to be actively canceled for the *non-working* finger (Table 2).

The functional connectivity between homologous areas of the right and left motor cortex has been studied using transcranial magnetic stimulation (TMS). The protocol employed is known to evoke interhemispheric inhibition (e.g., Nelson et al., 2009). During unimanual contractions the bi-directional interhemispheric inhibition is reduced bi-directionally across motor hemispheres and thus offers an explanation for the observation of ipsilateral motor cortex activity during hand movements (Nelson et al., 2009).

Analyzing event-related optical signals, Desoto et al. (2001) were able to confirm the hypothesis that conflicting trials activate both motor cortices simultaneously whereas non-conflicting trials evoke brain activity in the contralateral motor cortex only. Early bilateral cortex activation is in agreement with the current study, and is shown clearly in Figure 3 with a different timing for match trials (red) and the delayed mismatch (blue) trials. Moreover, an early inhibitory process must be assumed within the motor system to cancel the activation of the “wrong index finger” (Figure 5). From theoretical views it is assumed that the anterior cingulate cortex is probably involved in monitoring conflict conditions whereas the conflict itself is assumed to occur in different motor structures associated with competing responses (Carter et al., 1998; Gehring and Knight, 2000).

## Conclusion

The assumption that the negativity N0 corresponds with the end of a presumed deliberative process was derived from the comparison between EEG activities recorded during simple motor tasks and color-word deliberation tasks. The simple motor task was characterized by the subjects' awareness of the index finger to be employed to respond adequately to the upcoming stimulus whereas subjects were unaware of the finger to be employed during color-word deliberation tasks. Although the index finger movement itself in performing a keypress was identical during both tasks, the latency between the onset of the stimulus and the time of keypress was clearly longer during the deliberation tasks. A cortical potential sequence consisting of P1-N1-P2 components was observed during both tasks and thus was assumed to be related to the processing of the required movement. Even if a temporal jitter in the latencies was observed, N1 was closest to the time of keypress. Moreover, during deliberation tasks these sequences were found to be similar but superimposed on a preceding, slowly increasing negative potential, termed N0, during both match or mismatch-trials.

Due to the fact that the N0 was observed clearly prior to the sequence mentioned above, we assume that the N0 represents the end of the deliberation of the different stimuli. The length of the calculated onset of the slowly increasing slope to the N0 may reflect the duration of the deliberation process. Since the N0 was observed to be bilateral, but earlier in the ipsilateral motor areas, an interhemisperical and/or direct inhibiting process has to be assumed for canceling the activation for the non-working hand.

### Conflict of interest statement

The authors declare that the research was conducted in the absence of any commercial or financial relationships that could be construed as a potential conflict of interest.
